# Lifestyle and Diet as Risk Factors for Urinary Stone Formation: A Study in a Taiwanese Population

**DOI:** 10.3390/medicina59111895

**Published:** 2023-10-26

**Authors:** Ya-Chun Wu, Chen-Pang Hou, Shu-Chuan Weng

**Affiliations:** 1 Department of Healthcare Management, Yuanpei University of Medical Technology, Hsinchu 300, Taiwan; alice06090609@gmail.com (Y.-C.W.); glucose1979@gmail.com (C.-P.H.); 2 Hsinchu Branch, Taipei Veterans General Hospital, Hsinchu 310, Taiwan; 3 Department of Urology, Chang Gung Memorial Hospital at Linkou, Taoyuan 333, Taiwan

**Keywords:** urinary stone, lifestyle, diet, exercise

## Abstract

*Background and Objectives*: Urinary tract stones have long been a common ailment afflicting the population, with a high incidence and a wide distribution across different age groups. Effectively preventing the occurrence of urinary tract stones is of paramount importance. The primary aim of this study is to investigate the correlations between individual characteristics, water consumption habits, dietary habits, exercise habits, and the occurrence of urinary tract stones in a Taiwanese population. *Materials and Methods:* This study is cross-sectional research conducted over one month in 2022. One hundred eligible urinary stone cases were recruited through physician screening at outpatient clinics, and an additional one hundred samples from surgical outpatients without urinary tract stones were included as the control group. A questionnaire survey was employed to gather information on demographic variables, dietary habits, water consumption, and exercise habits of the cases. Descriptive statistics, chi-square tests, and logistic regression were used to explore the impact of relevant factors on urinary stone formation. *Results:* The analysis results revealed that among the demographic variables, males exhibited a higher risk of contracting urinary tract stones than females, and the majority of cases fell within the 40- to 49-year-old age group. Unhealthy lifestyle habits such as smoking and betel nut chewing also demonstrated a higher susceptibility to urinary tract stones. A logistic regression analysis showed that individuals who engaged in physical activity more than three times per week and those with inadequate water intakes had a higher risk of developing urinary tract stones. *Conclusions:* There is a close relationship between lifestyle and urinary tract stones. It is recommended that individuals continue to hydrate adequately during exercise.

## 1. Introduction

Urinary tract stones are prevalent disorders within the urinary system that occur in areas such as the kidneys, ureters, bladder, and urethra. The global prevalence is approximately 12%, with increasing rates in developed countries over the years. If not properly managed, urinary tract stones can lead to end-stage kidney disease [1,2]. The most common composition of these stones is calcium stones, accounting for around 60–80% of all types, followed by infection-related struvite stones and uric acid stones [3]. Clinical manifestations of stone-related symptoms are often unrelated to the stone’s composition and include symptoms such as flank pain, nausea, vomiting, fatigue, lower urinary tract symptoms, and hematuria [4].

In Taiwan, the prevalence of urinary tract stones is around 7.4–9.6%, with higher rates in males than females [5,6]. According to Huang et al. in 2013 [5], the occurrence in males is approximately 1.32–1.95 times that in females, with the peak incidence occurring between 60 and 69 years of age. The recurrence rate within one year is 6.12%, and the five-year recurrence rate is as high as 34.71%. Urinary tract stones are a common ailment in urology outpatient clinics, and they are equally prominent alongside benign prostatic hyperplasia and urinary tract infections as the main treatment concerns in urology outpatient care. Furthermore, the prevalence of urinary tract stones continues to rise. Survey results conducted among urologists from 27 countries have indicated that the most frequently treated conditions by urologists are urinary tract stones (30%), benign prostatic hyperplasia (15%), and prostate cancer (13%) [7]. Given the considerable volume of individuals seeking medical care for urinary tract stones and the propensity for recurrence, this places a substantial strain on healthcare resources [8].

Risk factors for urinary tract stones are multifactorial, including demographic factors, genetic inheritance, dietary habits, water intake, geographic location, seasonal variation, and disease-related factors [9]. Children with parents who have a history of urinary tract stones are more than three times as likely to develop stones [10]. Inadequate water intake, excessive consumption of dairy products and vitamin D, high intake of animal protein, refined carbohydrates, and salt are dietary factors contributing to stone formation [11,12]. Geographic location and season are related to sweating patterns, leading to decreased or concentrated urine production and stone formation [1]. In Taiwan’s subtropical climate, especially during the hot and sweaty summer months, the chances of stone formation are higher. Prolonged bed rest, conditions like hyperparathyroidism and hyperuricemia, and diabetes are also risk factors for urinary tract stones [13]. The first step in preventing urinary tract stones is increasing water intake, aiming for at least 2000 cc of water daily, consuming more fruits and vegetables, and avoiding holding urine to reduce the risk of urinary tract infections [14].

Taiwan is located in the subtropical region, and it has a high prevalence of urinary tract stones. Considering the rising trend in urinary tract stone incidence and increasing recurrence rates, Straub and Hautmann [12] stated that 85% of cases could reduce their risk through lifestyle and dietary adjustments. However, in the past, there has been a lack of research in Taiwan specifically examining the relationship between diet, lifestyle, and urinary tract stones. This study aims to analyze the impact of individual characteristics and lifestyle habits on urinary stone formation, with the goal of proposing feasible ways to mitigate stone formation risk through lifestyle changes.

## 2. Methods

### 2.1. Sample

This study commenced after obtaining approval from the Institutional Review Board for Human Research and Human Behavioral Research (protocol number YPU-IRB-1110816). From September 20th to October 2022, a total of 100 participants who met the criteria for urinary tract stones were screened by physicians at the urology outpatient department of a hospital. This hospital serves as the primary healthcare facility in Hsinchu County, located in the northern region of Taiwan. The inclusion criteria encompassed individuals aged between 20 and 70 years, possessing communication abilities, and having been diagnosed with urinary tract stones by specialized urologists in the hospital’s urology department. Pregnant women, due to potential differences in their lifestyle and diet during pregnancy, were also excluded from the study sample. Following an explanation of the research’s objectives and methods, an anonymous questionnaire survey was conducted. To compare the differences in lifestyle and dietary habits between individuals with and without urinary tract stones, we recruited additional cases without urinary tract stones from the same hospital. Since urinary tract stones are more likely to occur in young and middle-aged adults, this study recruited 100 control group participants aged between 20 and 70 years from a surgical outpatient department that primarily serves non-geriatric patients.

### 2.2. Research Instruments

The research employed a questionnaire survey as the research tool, using a self-designed questionnaire. The questionnaire covered various topics, including basic demographic information (such as gender, age, educational level, employment status, and habits like smoking, alcohol consumption, and betel nut chewing within the past year), family history of urinary tract stones, dietary habits, water consumption habits, and exercise habits. In terms of dietary habits, the questionnaire inquired about preference and frequency of consuming meat, seafood, dairy products, fried foods, fruits, and vegetables in the past year. Water consumption habits inquired about water sources and average daily water intake. Exercise habits involved questions about exercise frequency, intensity, and type.

The questionnaire used in this study was designed by the researchers, and it was primarily based on past research that identified lifestyle and dietary factors related to urinary tract stone formation. It aimed to inquire about the actual conditions of the participants. The questionnaire underwent expert content validation, where experts individually assessed the correctness, appropriateness, necessity, coverage of content, and accuracy of wording in the questionnaire and provided feedback. The final formal questionnaire had an overall content validity index (scale-level CVI, SCVI) of 0.81, indicating good validity. In the sections related to dietary habits, water consumption habits, and exercise habits, the Cronbach’s α values ranged from 0.69 to 0.81 based on a sample of 20 individuals, demonstrating good reliability.

The principal investigator provided explanations about the research objectives and procedures to the participants during recruitment. Upon obtaining consent, the paper-based questionnaire survey was conducted on-site. After confirming their completeness, the questionnaires were collected. The overall survey duration was approximately 10–15 min.

### 2.3. Statistical Analysis

This study utilized the Chinese version of SPSS 23 for Windows as the statistical software tool. After coding and quantifying the questionnaire, descriptive statistical analysis was conducted on the sample data using frequencies and percentages. Subsequently, chi-square tests were performed to examine whether there were differences in demographic variables, dietary habits, water consumption habits, and exercise habits between individuals with and without urinary tract stones. Lastly, binary logistic regression analysis was employed to investigate whether demographic variables, dietary habits, water consumption habits, and exercise habits of individuals with and without urinary tract stones would increase the risk of urinary stone formation. The model used a forward-conditional method for variable selection. The significance level for the study was set at 0.05.

## 3. Results

### 3.1. Descriptive Statistics of Basic Demographic Characteristics

During the study period, the research sample was recruited from the urology and surgical outpatient departments of the hospital. All individuals who met the criteria were asked if they were willing to participate in the survey until a total of 100 participants were enrolled in both the case and control groups. In total, there were 200 participants in the study sample. The results regarding the participants’ basic characteristics indicated the following: among the gender distribution, there were 129 males (64.5%); the age group of 40–49 had the highest frequency with 57 individuals (28.5%); in terms of educational attainment, 99 participants (49.5%) had completed university education; 129 participants (64.5%) were engaged in full-time employment; 159 participants (79.5%) identified as Hakka ethnicity; in the past year, the majority were non-smokers (59%); for alcohol consumption in the past year, 110 participants (55%) reported not consuming alcohol; and in terms of betel nut chewing in the past year, 178 participants (89%) reported not chewing betel nuts. Please refer to Table 1 for details.

### 3.2. Differences in Demographic Variables between Those with and without Urinary Tract Stones

This study was divided into two groups, comparing individuals without urinary tract stones and those diagnosed with urinary tract stones in terms of demographic variables, as shown in Table 2. In terms of gender, among individuals without urinary tract stones, 51% were female and 49% were male, while among those diagnosed with urinary tract stones, 20% were female and 80% were male. The chi-square test results indicated a statistically significant difference in gender distribution between those with and without urinary tract stones (χ^2^ = 20.985, *p* < 0.001).

Regarding age distribution, the chi-square test results showed a statistically significant difference in age distribution between those with and without urinary tract stones, with a higher proportion of older individuals being diagnosed with urinary tract stones (χ^2^ = 30.853, *p* < 0.001).

For educational attainment, the chi-square test results demonstrated a statistically significant difference in educational distribution between those with and without urinary tract stones, with a higher proportion of individuals with lower education levels being diagnosed with urinary tract stones (χ^2^ = 10.759, *p* = 0.029).

Concerning unhealthy lifestyle habits, the chi-square test results revealed a significantly higher proportion of smokers among those diagnosed with urinary tract stones (χ^2^ = 10.004, *p* = 0.013). Among individuals without urinary tract stones, 58% had never consumed alcohol, and 42% had consumed alcohol. The chi-square test results for betel nut chewing habits indicated a statistically significant difference in distribution between those with and without urinary tract stones (χ^2^ = 7.354, *p* = 0.007). Thus, there was a statistically significant difference in smoking and betel nut chewing habits as unhealthy behaviors.

Regarding urinary stone family history, the chi-square test results indicated a statistically significant difference in family history distribution between those with and without urinary tract stones (χ^2^ = 38.826, *p* < 0.001).

### 3.3. Analysis of Differences in Diet, Fluid Intake, and Exercise Habits between Those with and without Urinary Tract Stones

The chi-square test results indicated a statistically significant difference in the frequency distribution of consuming vegetables, seafood, soy products, and dairy products between those with and without urinary tract stones. Lower frequency of consuming vegetables was associated with a higher proportion of individuals diagnosed with urinary tract stones (χ^2^ = 27.917, *p* < 0.001); lower frequency of seafood consumption was associated with a higher proportion of individuals diagnosed with urinary tract stones (χ^2^ = 24.119, *p* < 0.001); lower frequency of soy product consumption was associated with a higher proportion of individuals diagnosed with urinary tract stones (χ^2^ = 32.199, *p* < 0.001); and lower frequency of dairy product consumption was associated with a higher proportion of individuals diagnosed with urinary tract stones (χ^2^ = 32.129, *p* < 0.001).

In terms of water consumption, it was found that individuals diagnosed with urinary tract stones had lower daily water intake (χ^2^ = 18.190, *p* < 0.001). However, no statistically significant differences were observed in water source (tap water) or water type (plain water).

Regarding exercise habits, only exercise frequency was found to have a difference in relation to the presence of urinary tract stones. When distinguishing between individuals with and without exercise habits, the chi-square test results indicated that those without exercise habits had a higher risk of being diagnosed with urinary tract stones (χ^2^ = 8.849, *p* = 0.013). However, when differentiating exercise frequency as more than 3 times per week and 3 times or less per week, the results of a univariate logistic regression indicated that compared to those who exercised 3 times per week, individuals who did not exercise at all had a lower risk of being diagnosed with urinary tract stones, although this difference was not statistically significant (OR = 0.306, *p* = 0.089).

### 3.4. Risk Analysis of Urinary Stone Formation

As shown in Table 3, this study included factors with significance values < 0.1 in the examination of their impact on urinary stone formation in a multivariate logistic regression analysis. The forward conditional method was employed for variable selection. The results revealed that only exercise frequency and daily water intake would affect the risk of urinary stone formation. When controlling for daily water intake, the group with minimal exercise had a smaller risk of urinary tract stones compared to those who exercised more than three times a week (OR = 0.164, *p* = 0.026). When controlling for exercise frequency, individuals with a daily water intake less than 1000 mL had a significantly higher risk of urinary stone formation, with the risk being as high as 15.989 times, particularly when compared to those with a daily water intake exceeding 2000 mL (*p* = 0.035).

## 4. Discussion

This study revealed significant associations within the demographic variables of the presence or absence of urinary tract stones. These included factors such as gender, age distribution, smoking habits, and urinary stone family history. Stern et al. [15] found that demographic variables such as age, gender, and ethnicity significantly impact health-related quality of life. They also noted that younger females with urinary tract stones have a better health-related quality of life compared to older adults and male patients. These demographic factors could influence aspects such as dietary education and follow-up health guidance for future urinary stone patients. Chewing betel nuts is a common habit among people in Southeast Asia, and it is considered alongside tobacco and alcohol as a harmful addictive substance. Previous studies have suggested that the use of calcium hydroxide in betel nuts, known as “chuna”, is a major cause of urinary stones in users [16]. However, this study did not find an association between betel nut consumption and urinary stones.

In the group with and without urinary tract stones, significant differences were observed in the frequency of consuming vegetables, seafood, soy products, and dairy products. According to D’Alessandro et al. [17], kidney stones are closely related to metabolic abnormalities and improper dietary habits. Increased intake of protein, particularly animal-derived protein, can lead to increased excretion of calcium, oxalates, and uric acid in the urine, contributing to the formation of kidney stones. While this study did not find a significant correlation between meat consumption frequency and urinary tract stones, it revealed that individuals diagnosed with urinary tract stones had lower frequencies of consuming vegetables, seafood, soy products, and dairy products, suggesting an unbalanced diet that might impact urinary stone formation.

Furthermore, the average daily water intake exhibited significant differences in the group with and without urinary tract stones. Bao, Tu, and Wei [18] conducted a year-long study that found increased water intake (more than 3000 mL per day) extended the time to recurrent stone formation for individuals with a history of urinary tract stones. Mitra, Pal, and Das [19] emphasized that water quantity, rather than quality, is crucial for preventing urinary tract stones, suggesting a daily intake of over 2000 mL of water. Theisen et al. [20] suggested that residents in arid areas who fail to replenish water in a timely manner could increase the risk of dehydration and, subsequently, urinary stone formation. Adequate water intake was shown to effectively reduce the incidence of urinary tract stones.

However, after including multiple variables in the regression model, only water intake and exercise frequency were found to be related to urinary stone formation, indicating that exercise habits and water intake indeed have a significant impact. The study found that individuals who exercised almost never had a smaller risk of urinary stone formation compared to those who exercised more than three times a week. This might be because exercisers tend to lose a significant amount of sweat after exercise, leading to higher urine concentration and potential stone formation. On the other hand, individuals who exercise less are at a lower risk of sweat loss. This suggests that exercise should be accompanied by proper hydration to avoid an increased risk of urinary stone formation. Aune, Mahamat-Saleh, Norat, and Riboli [21] found no significant association between exercise and urinary tract stones, and Jones et al. [22] highlighted excessive exercise and dehydration as risk factors for urinary stone formation, suggesting that excessive sweating from intense daily exercise for over 30 min increases the risk of urinary stone formation. This implies that excessive exercise leads to higher urine concentrations, making it easier for urinary tract stones to form. Thus, urinary stone patients should be advised to engage in moderate exercise and maintain proper hydration to support overall health.

The limitations of this study include the fact that it focused solely on patients from a single healthcare institution, which may result in regional variations. Additionally, the control group consisted of patients from the surgical outpatient department. While this choice helped mitigate the influence of other chronic conditions and ensured a closer age distribution to that of urinary tract stone patients, it is recommended that future analyses consider using healthy individuals as the control group. Besides, the research tool used in this study was a questionnaire survey. Apart from the potential recall bias among participants, as it was a self-designed questionnaire, although reliability and validity were tested, it cannot be directly compared with other studies. Furthermore, regarding the question related to the source of drinking water in the questionnaire, it was categorized into tap water, groundwater, spring water, and commercially bottled mineral water. Given that many households nowadays have water filtration systems, it is suggested that future research explore whether such filtration systems are installed.

## 5. Conclusions

There is a close relationship between lifestyle and urinary stone formation in the Taiwanese population. Maintaining healthy dietary habits, adequate water intake, and appropriate exercise may help prevent urinary stone formation. Even more importantly, it is essential to adequately hydrate before and after exercise to prevent excessive fluid loss, which may decrease the risk of urinary tract stones. Healthcare institutions should focus on preventing the recurrence of urinary tract stones, provide comprehensive and individualized education, and emphasize the importance of adequate hydration for patients with urinary stones.

## Figures and Tables

**Table 1:** Demographic data of samples.

Variable		n	%
Gender	Male	129	64.5%
	Female	71	35.5%
Age	20~29	28	14%
	30~39	25	12.5%
	40~49	57	28.5%
	50~59	49	24.5%
	60~69	41	20.5%
Educational level		3	1.5%
	Junior high school	23	11.5%
	Senior high school	69	34.5%
	College	99	49.5%
	Master	6	3.0%
Employment status	None	44	22%
	Part-time job	27	13.5%
	Full-time job	129	64.5%
Smoking within 1 year	Never	118	59%
	Quit already	22	11%
	Usually	45	22.5%
	Almost everyday	15	7.5%
Alcohol consuming Within 1 year	Never	110	55%
	Quit already	11	5.5%
	Usually	75	37.5%
	Almost everyday	4	2.0%
Betel nut chewing within 1 year	Never	178	89%
	Quit already	11	5.5%
	Usually	11	5.5%
	Almost everyday	0	0.0%

**Table 2:** Analysis of differences in basic demographics between those with and without urinary tract stones.

Variable		Without Urinary Tract Stones		With Urinary Tract Stones		χ^2^ *p*-Value	
		n	%	n	%		
Gender	Male	49	49.0%	80	80.0%	20.985	<0.001
	Female	51	51.0%	20	20.0%		
Age	20~29	25	25.0%	3	3.0%	30.853	<0.001
	30~39	15	15.0%	10	10.0%		
	40~49	30	30.0%	27	27.0%		
	50~59	20	20.0%	29	29.0%		
	60~69	10	10.0%	31	31.0%		
Educational level	Junior high school	9	9.0%	17	17.0%	10.759	0.029
	Senior high school	28	28.0%	41	41.0%		
	College	60	60.0%	39	39.0%		
	Master	3	3.0%	3	3.0%		
Employment status	None	23	23.0%	21	21.0%	3.471	0.176
	Employed	77	77.0%	79	79.0%		
Smoking	Never	70	70.0%	48	48.0%	10.004	0.002
	Yes	30	30.0%	52	52.0%		
Alcohol consuming	Never	58	58.0%	52	52.0%	0.727	0.394
	Yes	42	42.0%	48	48.0%		
Betel nut chewing	Never	95	95.0%	83	83.0%	7.354	0.007
	Yes	5	5.0%	17	17.0%		
Family history of urinary tract stones	No	47	78.3%	9	18.4%	38.826	<0.001
	Yes	13	21.7%	40	81.6%		

**Table 3:** Risk Analysis of Urinary Stone Formation.

	Adjusted OR	*p*-Value
Intercept	0.253	0.028
Exercise Habits (Ref. more than 3 times per week)		0.010
Almost no exercise	0.164	0.026
Less than twice per week	1.637	0.369
Fluid intake per day (Ref. more than 2001 mL)		0.036
Less than 1000 mL	15.989	0.035
1000~1500 mL	6.906	0.010
1501~2000 mL	4.008	0.023

## Data Availability

The data used to support the findings of this study are available from the corresponding author upon request.
